# Multicentre study of the role of lumbar puncture in the diagnosis of spontaneous subarachnoid haemorrhage

**DOI:** 10.1007/s00701-025-06602-8

**Published:** 2025-07-17

**Authors:** Daniel Thompson, Sara Venturini, Peter C. Whitfield, Peter Hutchinson, Nihal Gurusinghe, Rikin Trivedi, Adel Helmy, the LP for SAH diagnosis research collaborative, Peter A Bodkin, James LM Loan, Alexandria Habiby, Jack Latimer, Ibrahim Ibrahim, Bilal Benzahia, Neil Simms, Jamie Walsh, Theresa McGarry, Emer Moore, Mario Teo, Olivier Sluijters, Shammael Selorfia, Jahangir Sajjad, Shuaib Khan, Krunan Patel, Hassan Othman, Leevette Bay, Kathy Reeve, Muhammad Ammar Ali, Ahmed Abd Elmohseen, Mohsen Javadpour, Laura Kehoe, Deidre Nolan, Paula Corr, Deirdre Coffey, Mohamed Okasha, Victoria Collins, Michael Helley, Mark Highes, John-Paul Haugh, Molly Brewster, Samih Hassan, Allan Hall, Mustafa El Sheikh, Brian Ou Yong Ming, Ajitha Kulasekaran, Devansh Mitesh Shah, Abbie Simpson, Mihai Danciut, Edward Goacher, Asim Sheikh, Fozia Saeed, Elliot Tilling, Michael D Kenkinson, Ahmad M. S. Ali, Tarek Elmoslemany, Imran Farhad, Saniya Ansari, Nuzhat Choudhury, Aaditya Gurjer, Anjum Aarifa Khanom, Arun Rupra, Nigel Mendoza, Puneet Sharma, Ramesh Nair, Yi Wang, Gloria Quansah, Lydia Parker, Mohammad Al Hajjieh, Ahilan Kailaya-Vasan, James Knight, Proma Dey, Fatmah Noureldin, Patrick Grover, John Gerrard Hanrahan, Hamza A Salhab, Marwan Ali, Jamail Bal, Sobiya Bilal, Simon Williams, Steven Tominey, Abdalla Mansour, Fay Greenway, Natalia Simon, Gayathri Arul Selvan, Anindya Bhowmik, Eva Sherin Saravana Kumar, Nitin Mukerji, Nithish Jayakumar, Ekene Nnamani, Rasha Rajiv, Laurence Glancz, Natalia Rybka, Ananyo Bagchi, Andrew Edwards-Bailey, Samir Matloob, Jash Patel, Abdalla Mansour, Catherine Lamb, Nethmini Rathnayake, Dom Mahoney, Boh Sofela, Andrew Alade, Abdurrahman Islim, Jing Xian Lee, Mohammed Ibrahim, Swetha Joju, Andy Bacon, Matthew Sanders, Jerry Philip, Diederik Bulters, Samuel Hall, Emily Bligh, Soham Bandyopadhyay, Vishnu Suresh, Howard Brydon, Craig Robson, Rosa Sun, Anna Vassilou

**Affiliations:** 1 Department of Clinical Neurosciences, University of Cambridge, Cambridge, UK; 2 Department of Neurosurgery, University Hospitals Plymouth NHS Trust, Plymouth, UK; 3 Department of Neurosurgery, Royal Preston Hospital, Preston, England

**Keywords:** Lumbar puncture, Subarachnoid haemorrhage, Cerebral aneurysm

## Abstract

**Objectives:**

This study identified the proportion of spontaneous subarachnoid haemorrhage (SAH) patients diagnosed by Lumbar Puncture (LP). Furthermore reporting the incidence of aneurysmal SAH if a CT scan performed within 6 h was reported as negative, and finally investigated if there has been a change in practice since the new NICE guidance for the diagnosis of SAH was published in November 2022.

**Methods:**

A pragmatic multicentre audit was conducted in the UK and Ireland capturing referrals to 25 Neurosurgical centres between 1st November 2020—31st October 2023. Case referral identification was done in each unit using local medical records and referral databases based on local protocols.

**Results:**

10,187 cases of spontaneous SAH were diagnosed within the study period: 9,357 were diagnosed by CT and 717 by LP. 7% of all confirmed SAH cases underwent lumbar punctures to return a diagnosis of spontaneous SAH when a CT head scan was non-diagnostic. This yielded 213 (3%) diagnoses of aneurysmal SAH. 55 cases(1%) of aneurysmal SAH initially had negative CT head scans within 6 h of ictus and a positive LP. We did not identify any evidence of a change in practice following the introduction of the NICE guidance in November 2022.

**Conclusion:**

This study shows that LP continues to be an important diagnostic test that will confirm a diagnosis of aneurysmal SAH in a small, but significant number of patients with thunderclap headache. We provide new data that may impact the current NICE guidelines on the diagnosis of SAH.

## Introduction

Spontaneous subarachnoid haemorrhage (SAH) presents with a sudden onset severe headache and requires urgent investigation to identify an underlying cause and proceed to a potentially life-saving intervention if caused by an underlying cerebral aneurysm [14]. SAH caused by the rupture of an intracranial aneurysm is a Neurosurgical emergency and demands rapid diagnosis and management to improve patient outcomes and reduce avoidable morbidity and mortality [8]. In most cases SAH can be diagnosed using an unenhanced computer tomography (CT) scan of the brain, with high sensitivity for the presence of blood in the subarachnoid space [16]. However, there are cases where patients present to the Emergency Department with a history suggestive of SAH but where the CT scan does not show, or is not reported to show, blood in the subarachnoid space [2], termed Fisher 1 SAH [6]. Prior to the 2022 National Institute for Health and Care Excellence (NICE) Guidelines the management algorithm in these cases necessitates that a lumbar puncture (LP) is performed in order to test for the presence of bilirubin and oxyhaemolgobin within the cerebrospinal fluid (CSF) using spectrophotometery before specialised imaging investigations for the presumptive aneurysm [4, 18].

The National Stroke Guidelines recommended performing a LP in cases with a clinical history of a “sudden severe headache with an altered neurological state” if a CT head is negative [10]. The Guidelines do not make comment about the requirement for a CT head scan in a patient without neurological signs, advising that a LP may be necessary. The NICE Guideline (2022) stated that a sudden severe headache typically peaking in intensity within 1 to 5 min is a red-flag symptom of SAH. The guideline stated that “if a CT head scan done within 6 h of symptom onset and reported and documented by a radiologist shows no SAH (1) do not routinely offer a LP; (2) think about alternative diagnoses and discuss with a senior clinical decision maker; (3) seek advice from a specialist [11]. The NICE committee cited the invasive risks of LP and the cost as reasons not to routinely offer this gold-standard diagnostic procedure. The evidence that this recommendation was based on was graded as Moderate to Very Low by the authors [1, 3, 9, 12, 13]. This has led to concerns being raised by experts in vascular neurosurgery within the Society of British Neurological Surgeons (SBNS) and the British Neurovascular Group (BNVG), because of the risk of missing cases of SAH due to a ruptured intracranial aneurysm as a result of misinterpretation of an initial CT head scan or an occult haemorrhage. The standard/quality of CT head scan reports from most referring district hospitals is not that of an expert neuroradiologist.

We therefore convened a pragmatic review of the diagnosis of spontaneous SAH cases in order to ascertain how many are diagnosed by LP after a negative CT scan within 6 h of ictus in order to quantify the number of missed diagnoses that this change in recommendation could lead to. The primary aims of this service evaluation were to, firstly, identify the number of SAH cases that are diagnosed with a lumbar puncture across the UK, secondly to identify the number of aneurysmal SAH diagnoses made following a negative CT head scan within 6 h of ictus and thirdly review whether there has been a change in practice with regards to LP performed in suspected SAH cases since the publication of the new NICE guidance in November 2022.

## Methods

### Study design

This was a retrospective multicentre audit conducted in neurosurgical units in the United Kingdom (UK) and the Republic of Ireland (ROI). Participating units formed local teams to collect retrospective patient data from medical records and digital neurosurgery referral databases. Only routine, anonymised data were collected and centrally analysed. No clinical care pathways were changed.

### Study setting and approvals

The audit was conducted in neurosurgical units across the UK and ROI. All neurosurgery units accepting referrals for SAH were invited to participate. This was an audit project and collaborators at each participating site filed local approval in accordance with their service evaluation/audit department protocols.

### Population

All patients referred to a participating Neurosurgical centre for SAH and who had a confirmed diagnosis of spontaneous SAH during the study period were included in the study. Traumatic SAH cases were excluded. Cases diagnosed over a three-year period from 1 st November 2020 to 31 st October 2023 were included. Case referral identification was done in each unit using local medical records and referral databases based on local protocols. The use of different types of electronic health records and referral systems by different hospitals meant this process could not be standardised. In keeping with the pragmatic nature of this multicentre study, local protocols were used for the diagnosis of LP positive SAH. Two patient cohorts were formed: the patients diagnosed before 1 st November 2022, before the new NICE guidelines for SAH were proposed, and the patients diagnosed from 1 st November 2022 to 31 st October 2023, after the introduction of the new NICE guidelines.

### Data collection

Data collection was performed according to a standardised proforma. Collected data included a summary of total spontaneous SAH cases diagnosed at each participating site during the study period, with numbers identified for each month, and grouped according to diagnostic modality (CT, LP or other). In addition, more granular data were collected for those patients where the SAH diagnosis was confirmed via LP after an initially reported negative CT brain. These patients were grouped into a pre-guideline cohort and a post-guideline cohort. Data fields collected were: initial imaging timing and report (including any addenda to the report), details of when the LP was performed, its results, time to SAH diagnosis and final SAH diagnosis type.

### Statistical analysis

Individual centre data was combined into a single dataset for analysis. This dataset comprised all cases where data fields pertaining to LP diagnosis and final SAH diagnosis type (aneurysmal, non-aneurysmal) were complete. All analysis was performed using STATA/SE version 18.0. Diagnostic practice was analysed to investigate if the number of LP, CT head scan and other means of obtaining a diagnosis had altered as a result of NICE guidance. A Pearson Chi squared test of independence was performed. This was repeated to analyse whether a greater or lesser proportion of lumbar punctures were being performed on patients with negative CT head scans within 6 h. The time from ictus to LP and ictus to confirmed diagnosis are also reported using proportions.

### Patient and public involvement

This study did not involve patient or public involvement in its design, conduct, reporting, or dissemination.

## Results

### Overview

25 out of the 31 centres providing adult Neurosurgical care within the UK and Ireland submitted complete data (Appendix 1).

**Fig. 1:**
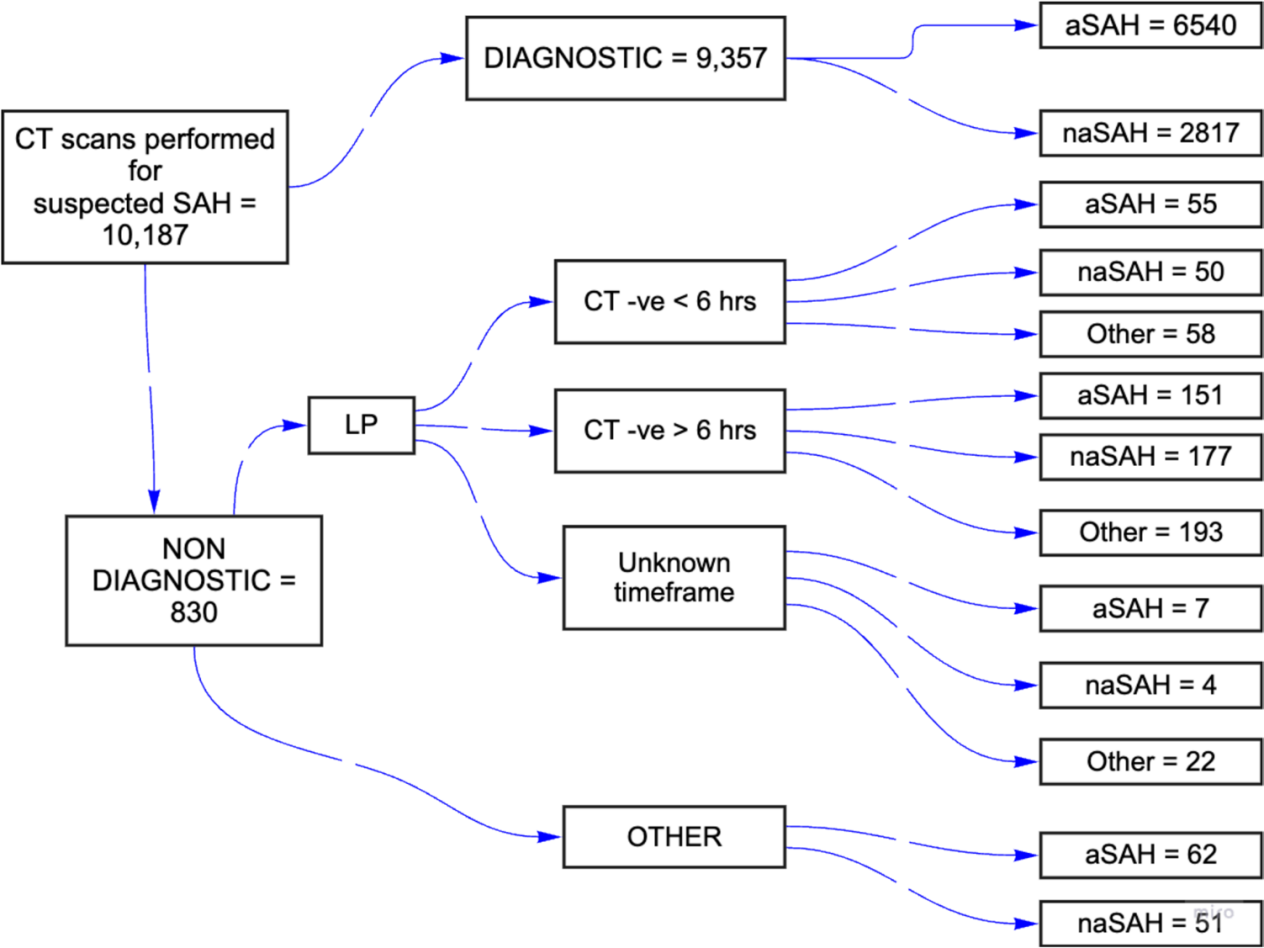
Flow chart demonstrating the overall number of patients included in the study as well as the diagnostic modalities and eventual diagnoses *aSAH = aneurysmal SAH, naSAH = non-aneurysmal SAH

The flow diagram in Fig. 1 demonstrates the method of diagnosis over the study period and the diagnoses. There were 10,187 cases of spontaneous SAH diagnosed and referred to participating centres. 6,815 (67%) were aneurysmal SAH and 3,372 (33%) of these were non-aneurysmal SAH, following dedicated neurovascular imaging. 9,357 (92%) of cases were diagnosed by CT head scan. 717 (7%) of cases were diagnosed using LP after a CT was reported as non-diagnostic for SAH. This led to the diagnosis of 213 aneurysmal SAH, which represents 3% of all diagnosed aneurysmal SAH.

In addition, 72 of the CT negative aneurysmal SAH cases (0.7% of total; 34% of CT negative aneurysmal SAH subgroup) were confirmed by an addendum radiology report indicating that SAH was in fact present on the initial scan even though it was incorrectly reported as negative. These numbers are included in the overall CT scan diagnosis group.

### The relationship between NICE guidance and diagnostic modality

The Pearson Chi-squared test of independence was used to see if NICE guidance had altered the proportions of different diagnostic modalities. Table 1 demonstrates the findings and suggested a relationship between diagnostic modality and guideline implementation, with there being more LPs and other tests confirming diagnosis following the guidance than would be expected.

**Table 1:** Referrals made for suspected SAH described by diagnostic modality and pre/post-NICE guidance change

Diagnostic modality		
	Pre-NICE (24 months)	Post-NICE (13 months)
CT	6318 (6275) [0.29]	3039 (3082) [0.59]
LP	454 (480) [1.50]	263 (236) [3.06]
Other	60 (76) [3.29]	53 (37) [6.69]
*Column totals*	**6832**	**3355**

Pearson Chi2(2) = 15.4187, *p*-value is < 0.001

() Shows expected frequency. [] Shows Chi2 value

### Positive LPs when CT head negative within 6 h post-ictus

Table 2 shows the number of LPs carried out in patients with a negative CT scan within 6 h of ictus and this does not demonstrate a relationship between NICE guidance implementation and the proportion of lumbar punctures being performed for CT head scans within or after 6 h. The Pearson Chi-squared test of independence does not deliver a statistically significant result.

**Table 2:** Comparison of the number of lumbar punctures performed described by timing of CT head and whether it was performed pre/post-NICE guideline change

	CT within 6 h post-ictus	
	No	Yes
Pre-NICE guidance	336 (77%)	99 (23%)
Post-NICE guidance	185 (74%)	64 (26%)
	**521**	**163**

Pearson Chi2(1) = 0.7562, *p* value = 0.385

There were 717 lumbar punctures performed during the study period. 163 (23%) of these were performed after a negatively reported CT head scan performed within 6 h of ictus and 521 (73%) were as a result of scans performed outside of this window. For 33 patients (5%) the timing of the CT scan was unknown.

Of the 163 CT head scans performed within 6 h and reported as negative, 55 were subsequently diagnosed as aneurysmal subarachnoid haemorrhage (aSAH), 50 as non-aneurysmal SAH (naSAH), and 58 were found to have an alternative diagnosis. Therefore in total 1% of the aneurysmal SAH cases diagnosed within the 3-year study period were confirmed by LP following a CT head scan that was reported as negative within 6 h post-ictus.

### Diagnosis following LP

**Fig. 2:**
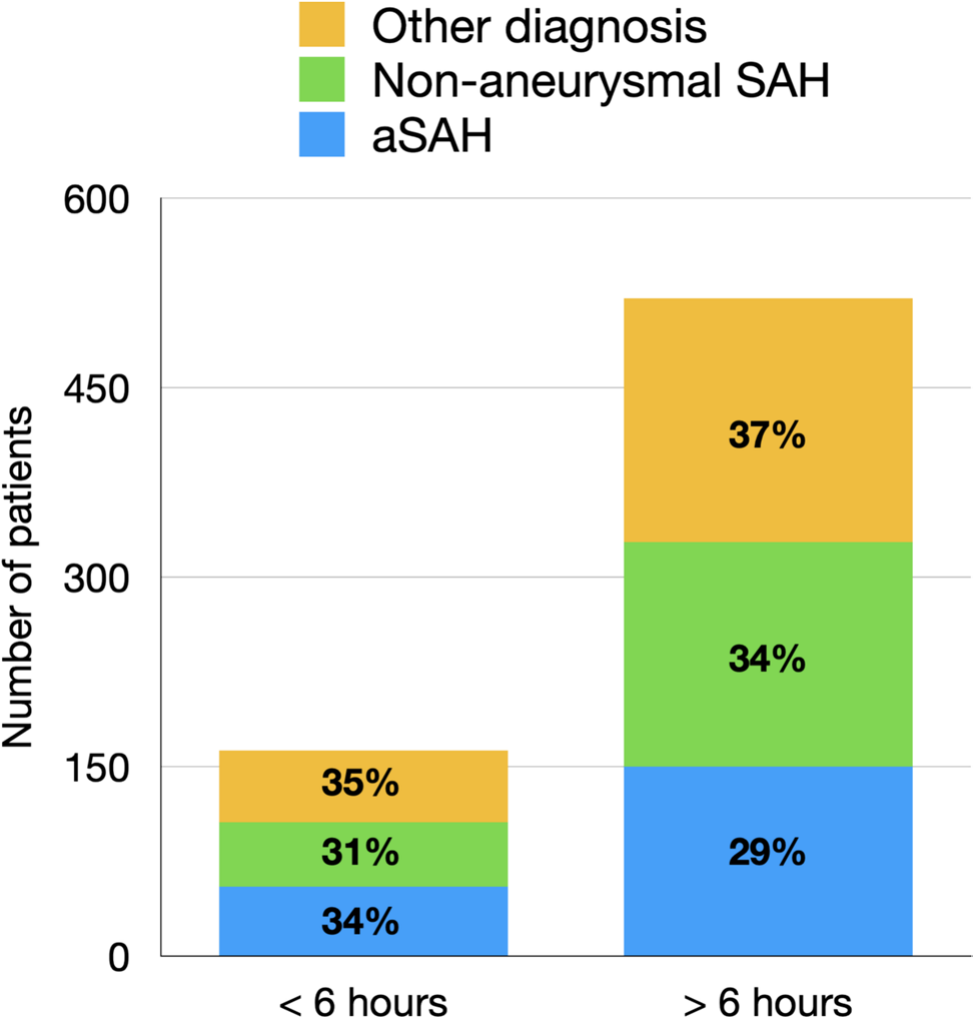
The diagnosis confirmed by LP for negative CT head scans within 6 h and after 6 h post-ictus

For patients undergoing lumbar puncture (717) following a negative CT head scan: 213 (30%) had aneurysmal SAH, 231 (32%) had a non-aneurysmal SAH and 273 (38%) had a different diagnosis. Figure 2 shows these by whether or not the negative CT head was performed before or after 6 h. Among patients with a negative CT head scan, those undergoing lumbar puncture within 6 h of ictus were diagnosed with aneurysmal SAH in 34% of cases, compared to 29% when the lumbar puncture was performed after 6 h.

## Discussion

The accurate and timely diagnosis of subarachnoid haemorrhage is of paramount importance to the Neurosurgical community and emergency services at large because of the potential to save lives. Our study captured referrals from the majority of Neurosurgical centres within the UK and Ireland. Overall, it demonstrated that 7% of all referrals made to Neurosurgical centres with a suspicion of spontaneous SAH underwent LPs as a part of their work up. Notably, 3% of all aneurysmal subarachnoid haemorrhage (aSAH) diagnoses were made following lumbar puncture. In addition, 1% of all aneurysmal SAH diagnoses had negatively reported CT head scans within 6 h post-ictus and a positive LP. Furthermore, 229 diagnoses of non-aneurysmal SAH bleeds were diagnosed via LP. While the risk of subsequent deterioration and death with non-aneurysmal SAH is much reduced compared to aneurysmal SAH, this is a diagnosis of exclusion and necessitates vascular neuroimaging, typically in a neuroscience specialist centre.

There were also 72 cases where an aneurysmal SAH was only diagnosed on a CT head scan following an addendum to the report, leading to an avoidable delay in diagnosis and therefore the speed of securing the underlying aneurysm. The risk of aneurysmal re-bleeding is highest within the initial 24 h of ictus [15], and avoidable delays should be minimised. We were unable to collect data on the average delay between which the report was released and the addendum was filed, however, it emphasises the difficulty with relying upon reports from General radiologists in the context of SAH. Indeed the American Heart Association/American Stroke Association 2023 SAH guidelines specifically mandate a board-certified Neuroradiologist to report a scan that can be classified as negative within 6 h of ictus [7].

NICE guidance was published on the 23 November 2022 regarding the diagnosis and management of subarachnoid haemorrhage [11]. Our study demonstrates that there has not been a statistically significant change in practice since it was circulated. It advises that patients presenting with symptoms consistent with SAH who undergo CT head scans within 6 h post-ictus of headache that are reported as negative for SAH should not routinely be offered a lumbar puncture. This view does not accord with the national neurosurgical body, the Society of British Neurological Surgeons (SBNS), who have raised concerns that some patients with a potentially life-threatening and treatable diagnosis will be under-investigated because of this guidance. CT head scans are not 100% sensitive at detecting SAH and errors in the reporting of scans were not considered by NICE when publishing the guideline. The new guidance was predominantly based on evidence from seven papers that investigated the sensitivity and specificity of the diagnostic modality of CT head scans for suspected SAH [1, 2, 9, 12, 13]. The papers represent a heterogenous group with study sizes varying between 155 patients to 3,132. The largest study described the overall sensitivity of CT head scans in the diagnosis of SAH as 92.9% [12]. A smaller retrospective study concluded that the sensitivity of CT head within 12 h was 95%. However, this same study concluded that although highly sensitive CT is not sufficient as a sole diagnostic tool and that those with a high clinical suspicion should undergo lumbar puncture following a negative CT head [13].

A meta-analysis by Dubosh et al. regarding the sensitivity of CT scans within 6 h of ictus demonstrated a sensivity of 99% and concluded that this may be sufficient to rule our SAH in a neurologically normal patient with a thunderclap headache [5]. However, in their heterogenous study group 2 out of the 5 studies had a “miss rate” of over 1%, which is the finding within our study [1, 9]. The study by Mark et al. where the “miss rate” may have been as high as 20% was also one within which General Radiologists read the initial reports and not Neuroradiology specialists. This speaks to the concern that academic studies relying on expert review of CT scan by one or more radiologists, who are specifically ‘primed’ to identify subarachnoid haemorrhage, is not representative of the on-call diagnostic imaging services provided in most National Health Service (NHS) settings. Out of hours imaging may be reported by trainee/resident radiologists, and the pressure of time and volume of reporting in an on-call setting increases the chances of subtle imaging findings being missed.

Spontaneous SAH is a potentially life-threatening condition that requires urgent Neurosurgical management to help prevent a poor outcome. Although the cohort being considered in the arm of the study that required LP for diagnosis is a more medically stable population it is still imperative that those with a World Federation of Neurosurgical Societies (WFNS) Grade I SAH are diagnosed and managed efficiently, as they have the most to lose from a re-bleed in terms of morbidity and mortality [15]. Our study revealed that 1% of patients could be at risk of late diagnosis and the possible devastating sequelae that this entails if the clinical history is ignored. Furthermore, the risk of SAH being missed by the initial report and only identified via an addendum may further inflate this figure. We believe the strength of our study, in contrast to previous work, lies in its real-world setting, offering a pragmatic context in which to assess the validity of enforcing a diagnostic framework when the underlying evidence remains hotly debated (Fig. 2).

This study has several limitations that should be acknowledged. Firstly, it is a retrospective study and therefore prone to missing data. Importantly, the study protocol did not dictate a specific method to identify SAH cases diagnosed during the study period, because of the heterogenous nature of referral systems and electronic healthcare records. Each participating site utilised their own neurosurgical referral system and chart review to identify eligible patients. This means that a minority of cases could have been missed, for example cases where there was no written referral documentation, or cases where a patient was never referred to a neurosurgical unit. However, most neurosurgical units in the UK now utilise electronic referral systems such as Orion or Referapatient, which allow for case screening, which should limit the number of missed cases. The study also only captures those patients that were referred to a Neurosurgical centre with a suspected SAH. This means it is not able to comment on overall referral numbers in Emergency Departments, nor the specificity or sensitivity of CT head scans in diagnosing this condition. Equally there may be centres where an LP is performed without informing the Neurosurgical oncall and thus the numbers of these may be an underestimate. Furthermore, we do not report upon the numbers of complications incurred by performing LP. However, due to the modest numbers reported in the literature we would not envisage these having an impact upon the overall message of our paper [17]. Despite these limitations, this pragmatic study collected data from the majority of neurosurgical units in the UK and ROI, likely being representative of the case mix seen and patient population.

The NHS is under considerable pressure from increasing population demands, an ageing population and an increasingly complex cohort of patients. These pressures are often concentrated in Emergency Departments and there is an understandable desire to streamline pathways and minimise unnecessary investigations. The authors of this paper, as representatives of the SBNS, would strongly advocate that current evidence continues to support the use of LP in patients with a clinical history suspicious for SAH, even with a reportedly negative CT scan within 6 h.

## Conclusion

1% of all aneurysmal SAH were diagnosed by LP having initially had negative CT head scans within 6 h of ictus. There does not appear to have been a change in practice when diagnosing suspected spontaneous SAH since the new NICE guidance was delivered in November 2022. This study shows that LP continues to be an important test that will secure a diagnosis of aneurysmal SAH in a small, but significant number of patients with thunderclap headache. This study provides new data that may impact the current NICE guidelines on the diagnosis of SAH.

## Data Availability

Full dataset that supports the findings of this study has been deposited at: 10.6084/m9.figshare.28890338.v1.

## Appendix 1: List of Hospitals participating in study

Aberdeen Royal Infirmary.

Beaumont Hospital, Dublin.

Cambridge University Hospitals.

Hull Royal Infirmary.

Imperial College Healthcare NHS Trust.

King’s College Hospital.

Leeds General Infirmary.

National Hospital for Neurology and Neurosurgery. Ninewells Hospital, Dundee.

Ninewells Hospital, Dundee.

North Bristol Trust.

Nottingham University Hospitals NHS Trust.

Oxford University Hospitals.

Queen Elizabeth University Hospital, Glasgow.

Royal Hallamshire Hospital.

Royal Infirmary of Edinburgh.

Royal Preston Hospital, Lancashire Teaching Hospitals NHS Foundation Trust.

Royal Stoke University Hospital.

Royal Victoria Hospital, Belfast.

South Tees Hospitals NHS Foundation Trust.

St George’s Hospital.

The Royal London Hospital.

The Walton Centre.

University Hospital Coventry & Warwickshire.

University Hospitals Plymouth NHS Trust.

University Hospital Southampton.
